# Biofilm and Planktonic Bacterial and Fungal Communities Transforming High-Molecular-Weight Polycyclic Aromatic Hydrocarbons

**DOI:** 10.1128/AEM.03713-15

**Published:** 2016-04-04

**Authors:** Benjamin D. Folwell, Terry J. McGenity, Corinne Whitby

**Affiliations:** School of Biological Sciences, University of Essex, Colchester, United Kingdom; University of Calgary

## Abstract

High-molecular-weight polycyclic aromatic hydrocarbons (HMW-PAHs) are natural components of fossil fuels that are carcinogenic and persistent in the environment, particularly in oil sands process-affected water (OSPW). Their hydrophobicity and tendency to adsorb to organic matter result in low bioavailability and high recalcitrance to degradation. Despite the importance of microbes for environmental remediation, little is known about those involved in HMW-PAH transformations. Here, we investigated the transformation of HMW-PAHs using samples of OSPW and compared the bacterial and fungal community compositions attached to hydrophobic filters and in suspension. It was anticipated that the hydrophobic filters with sorbed HMW-PAHs would select for microbes that specialize in adhesion. Over 33 days, more pyrene was removed (75% ± 11.7%) than the five-ring PAHs benzo[*a*]pyrene (44% ± 13.6%) and benzo[*b*]fluoranthene (41% ± 12.6%). For both bacteria and fungi, the addition of PAHs led to a shift in community composition, but thereafter the major factor determining the fungal community composition was whether it was in the planktonic phase or attached to filters. In contrast, the major determinant of the bacterial community composition was the nature of the PAH serving as the carbon source. The main bacteria enriched by HMW-PAHs were Pseudomonas, Bacillus, and Microbacterium species. This report demonstrates that OSPW harbors microbial communities with the capacity to transform HMW-PAHs. Furthermore, the provision of suitable surfaces that encourage PAH sorption and microbial adhesion select for different fungal and bacterial species with the potential for HMW-PAH degradation.

## INTRODUCTION

Polycyclic aromatic hydrocarbons (PAHs) are a diverse class of organic molecules that consist of two or more benzene rings in linear, angular, or cluster arrangements (1). PAHs with more than three aromatic rings are referred to as high-molecular-weight PAHs (HMW-PAHs). The U.S. Environmental Protection Agency (EPA) classified 16 PAHs as priority pollutants based on toxicity, potential for human exposure, and frequency of occurrence at hazardous waste sites. Seven of these, including benzo[*a*]pyrene (BaP) and benzo[*b*]fluoranthene (BbF), are regarded as probable human carcinogens (2). The European Union Water Framework Directive (EU WFD) has also identified HMW-PAHs as priority hazardous substances, naming five key indicator compounds, including both BaP and BbF (3). Generally, PAHs that are considered to be carcinogenic have a higher molecular weight and a lower solubility than noncarcinogens (2). These physico-chemical characteristics also contribute to their recalcitrance (4).

Although HMW-PAHs are present in oil sands in Alberta, Canada (5, 6), attention has focused on the acutely toxic naphthenic acids. Oil sands operations in Canada produce more than 200 million barrels of crude oil per year (7). During oil sand extraction, vast quantities of wastewaters known as tailings are generated, which have to be stored indefinitely in settling ponds until strategies for reclamation are devised and approved. Tailings are composed of solids (e.g., sand and silt) and oil sands process-affected water (OSPW). OSPW contains approximately 0.01 mg liter^−1^ of PAHs, a value 1,000-fold greater than the limit specified in the Canadian Environmental Quality Guidelines (8). Currently, very little is known about the composition and roles of microorganisms involved in HMW-PAH biodegradation. The ubiquitous coexistence of bacteria and fungi in soil and sediments and their known catabolic cooperation suggest that interactions between them may be important for PAH degradation (9). Considerable interest has arisen from using fungi in soil and sediment remediation processes due to the low number of bacteria that are known to degrade PAHs with more than four fused aromatic rings (10). Numerous ligninolytic and nonligninolytic fungi possess the ability to oxidize PAHs (11). Fungal exoenzymes have the advantage that they may diffuse to the highly immobile HMW-PAHs (12), avoiding the need for HMW-PAHs to enter the cell as required for bacteria with intracellular PAH-degrading enzymes. However, when the PAH is oxidized by fungal exoenzymes, further metabolism by bacterial enzymes may facilitate complete mineralization (13).

Biodegradation of HMW-PAHs is strongly affected by bioavailability, as their limited water solubility causes them to adsorb strongly to organic particles (14). However, in the laboratory, enrichment of microorganisms capable of HMW-PAH biodegradation has been done mostly in shaken liquid media. These conditions are very different from those experienced by microbes in natural environments where compounds are sorbed to organic matter on sediment particles. In this study, OSPW-containing microcosms in which PAHs were provided in a sorbed state to enrich for adhering HMW-PAH-degrading microorganisms were established. The reduction in distance between the microbial cells and the HMW-PAH increases bioavailability and thus accelerates biodegradation (14).

The aims of this study were to measure the biotransformation capacities of three sorbed HMW-PAHs in two different OSPW samples and to determine the bacterial and fungal community compositions in both the biofilm and planktonic phase during transformation. The three HMW-PAHs, pyrene (Pyr), benzo[*a*]pyrene (BaP), and benzo[*b*]fluoranthene (BbF), were selected based on differences in physico-chemical characteristics and toxicity (Table 1).

**TABLE 1:**
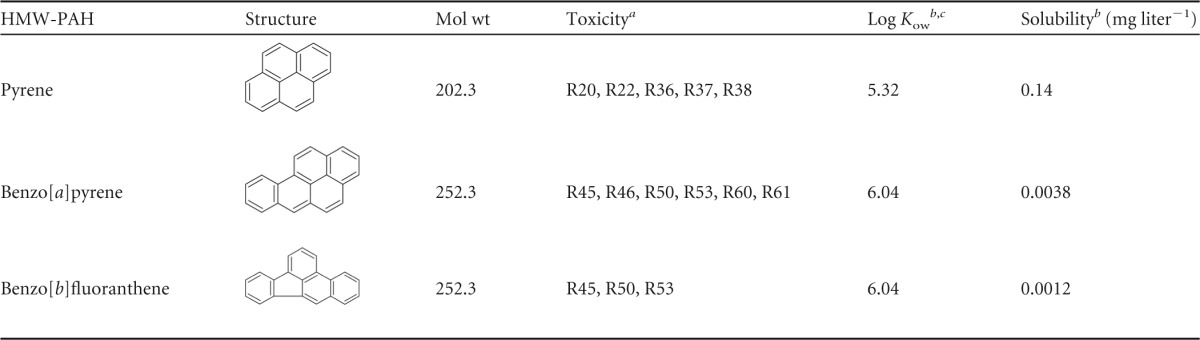
Structures and physico-chemical properties of target HMW-PAHs

a Toxicity codes are from Material Safety Data Sheet (MSDS) data. R20, harmful by inhalation; R21, harmful in contact with skin; R22, harmful if swallowed; R36, irritating to eyes; R37, irritating to respiratory system; R38, irritating to skin; R40, limited evidence of a carcinogenic effect; R45, may cause cancer; R46, may cause heritable genetic damage; R50, very toxic to aquatic organisms; R51, toxic to aquatic organisms; R53, may cause long-term adverse effects in the aquatic environment; R60, may impair fertility; R61, may cause harm to the unborn child; R68, possible risk of irreversible effects.

b Values are from reference 55.

c *K*_ow_, octanol-water partition coefficient.

## MATERIALS AND METHODS

### Environmental samples.

A tailings pond water sample (designated TPW) was collected from a Syncrude test pit (GIS 57.0380885, −111.5407123) courtesy of Warren Zubot (Syncrude). The test pit was excavated in 1993 and filled with aged recycled water from the Mildred Lake Settling Basin (MLSB). A second tailings pond water sample (designated 2m) was supplied by L. Gieg (University of Calgary) and collected at a water depth of 2 m from a Suncor tailings pond (GIS 56.9923073, −111.4979146).

For ion chromatography, environmental samples were diluted 10-fold in MilliQ water and filtered through a GF/F filter (Whatman) by vacuum. Samples were analyzed using an ICS-3000 Dionex. For cation analysis, the eluent was 20 mM methyl sulfonic acid run at a flow rate of 1 ml min^−1^ for 30 mins on a Dionex Ionpac 4-mm column (column temperature, 30°C). The eluted cations were detected with a CSRS 300 4-mm Suppressor onto a conductivity cell detector. For the anion analysis, the eluent was MilliQ water and 100 mM KOH. Potassium hydroxide was run on a gradient with a flow of 0.25 ml min^−1^. This was run through an Ionpac AS 18 2-mm column onto a conductivity cell detector. For total organic carbon (TOC) analysis, samples were measured using a Shimadzu TOC-VCHS pyrolyser fitted with an SSM 5000A solid sample module and running TOC-Control V software v. 2.00.

### HMW-PAHs.

Three high-molecular-weight polycyclic aromatic hydrocarbons (HMW-PAHs) were used in this study: pyrene, benzo[*a*]pyrene, and benzo[*b*]fluoranthene. All compounds were obtained from Sigma-Aldrich, Gillingham, United Kingdom (high-performance liquid chromatography [HPLC] grade; >98% purity for all compounds).

### Growth medium.

The growth medium used for all aerobic transformation studies contained, per liter, the following: MgSO_4_ · 7H_2_O, 0.5 g; CaCl_2_ · H_2_O, 0.1 g; NH_4_NO_3_, 1 g; Na_2_HPO_4_, 1.1 g; KH_2_PO_4_, 0.25 g, and trace elements, 1 ml. The trace elements included, per liter, the following: FeSO_4_ · 7H_2_O, 10 mg; Na_2_EDTA · 3H_2_O, 0.64 mg; ZnSO_4_ · 7H_2_O, 0.2 mg; H_3_BO_3_, 0.015 mg; CoCl_2_ · 6H_2_O, 0.175 mg; Na_2_MoO_4_, 0.14 mg; MnCl_2_ · 4H_2_O, 0.02 mg; and NiCl_2_ · 6H_2_O, 0.01 mg (adjusted to pH 7.0 and autoclaved). Four vitamin solutions were filter sterilized using a 0.2-μm filter and added to the autoclaved medium at 1 ml per solution. Vitamin solution 1 contained, per liter, the following: biotin, 50 mg; pantothenate, 50 mg; folic acid, 20 mg; lipoic acid, 50 mg; pyridoxine, 100 mg; and nicotinamide, 550 mg. Vitamin solution 2 contained 100 mg thiamine per liter, vitamin solution 3 contained 50 mg riboflavin per liter, and vitamin solution 4 contained 250 mg cyanocobalamin per liter. For isolation of colonies, the growth medium also contained 15 g liter^−1^ of bacteriological agar (Difco).

### HMW-PAH-transforming enrichment cultures.

Each HMW-PAH (10 mg), i.e., Pyr, BaP, and BbF (10 mg), was dissolved in 1 ml of acetone (Fisher Scientific), 100 μl of which was spiked onto a polytetrafluoroethylene (PTFE) filter (Fisher Scientific). Four PAH-containing filters were added as the sole carbon and energy source to each flask, giving a final PAH concentration of 200 mg liter^−1^. Prior to addition, filters were left for 1 h to allow the acetone to evaporate. Control filters containing acetone alone (100 μl) and abiotic controls containing the individual HMW-PAHs at 10 mg ml^−1^ dissolved in acetone and added to growth medium (final concentration, 200 mg liter^−1^) were also prepared. All cultures were incubated statically in the dark at 20°C for 8 weeks for the initial enrichment, and growth was monitored visually by turbidity. One of the four filters (denoted the enrichment filter) and 2% (vol/vol) of the planktonic phase were used to inoculate the second enrichment, which was then incubated for 6 weeks as described above. Two subsequent enrichments were then incubated for a further 4 weeks statically in the dark at 20°C.

PAH transformation experiments were set up as follows. Pyrene, BaP, and BbF were added (final concentration, 5 mg liter^−1^ dissolved in acetone) to 120-ml serum bottles. The bottles were then left uncapped in a laminar flow cabinet for 1 h to allow the acetone to evaporate. Serum bottles were then filled with 100 ml of growth medium and inoculated with one enrichment filter, either TPW or 2m, and 2% (vol/vol) of planktonic phase from the same enrichment culture, capped, and crimp sealed. For example, a filter and 2% (vol/vol) of the planktonic phase from a TPW sample enriched on Pyr was used for inoculation in the biotransformation experiment with TPW and Pyr. Killed controls (to determine if any abiotic loss had occurred) were also prepared by Tyndallization before HMW-PAH addition. Viability was checked by inoculating 100 μl of culture on LB plates and incubating at 20°C. Abiotic controls containing the HMW-PAH compounds only (final concentration, 5 mg liter^−1^) and procedural blanks containing either 2% (vol/vol) enrichment culture alone or 2% (vol/vol) medium alone (i.e., no culture and no PAH) were also prepared. All cultures were incubated statically at 20°C in the dark for 33 days. At each time point (0, 11, and 33 days), a 25-ml subsample was taken and then centrifuged (3,435 × *g*) for 10 min. The cell pellet and filters from day 33 of the biotransformation experiments were stored at −20°C prior to DNA extraction and subsequent community analysis.

### Isolation of HMW-PAH-utilizing cultures.

Either planktonic culture (100 μl) or a filter was taken from the fourth enrichment culture and placed on a growth medium agar plate containing 1 mg of the same HMW-PAH (e.g., a filter taken from a pyrene enrichment culture was placed on a plate containing 1 mg pyrene). For each HMW-PAH (Pyr, BaP, and BbF), 10 mg was dissolved in 1 ml of acetone (Fisher Scientific), 100 μl of which was spiked onto the plate surface. Prior to inoculation, plates were left for 1 h for acetone to evaporate. Plates were incubated at 20°C for 10 days before single colonies were selected and subcultured until pure colonies were obtained. Strains were subcultured up to five times on plates containing the target HMW-PAH as the only carbon source until pure colonies were obtained.

### Solvent extraction and quantification of HMW-PAHs.

All glassware was prepared as described elsewhere (15). The internal standard 2-methylnaphthalene (Acros Organics) (10 mg) dissolved in 1 ml of methanol (HPLC grade) was used (final concentration, 2 mg liter^−1^). All filters, along with the supernatants, were included in the solvent extractions to determine any residual PAH. Each PAH was extracted three times as described previously (15) (Fisher Scientific) using ethyl acetate (Fisher Scientific). Samples were resuspended in 1 ml of dichloromethane (HPLC grade; Acros Organics) and separated by gas chromatography-mass spectrometry (GC-MS) using an Agilent 7890 GC interfaced with an Agilent 5975C MS. Samples were injected with a 1-μl splitless injection (injector temperature, 250°C) onto an Rtx-1MS column (30 m by 250 μm by 0.25 μm) using helium as the carrier gas at a constant flow of 1 ml min^−1^. Oven temperatures were programmed with an initial increase from 40°C to 300°C at 10°C min^−1^ and a final hold at 300°C for 10 min. The transfer line was held at 230°C onto a source for the MS, which was in full-scan mode (scan range, 50 to 650 Da). Data were analyzed and integrated using ChemStation for GC-MS (Agilent), and the mass spectra of putative metabolites were identified by library comparison.

### DNA extraction and PCR amplification of bacterial 16S rRNA genes and the fungal intergenic transcribed spacer (ITS) region.

Total nucleic acids were extracted from the filters and planktonic cultures of the biotransformation experiments (day 33) as described previously (16). Cells were eluted from filters by suspension in 4 ml of lysis buffer (100 mM Tris-HCl, 50 mM NaCl, 50 mM EDTA, pH 8.0) and vortexing for 2 min. DNA was extracted from isolates as previously described by (16). PCR amplifications were performed using primers F341-GC and R534 (17) for bacteria and ITS3 with a GC clamp (sequence as for F341-GC) and ITS4 (18) for fungal denaturing gradient gel electrophoresis (DGGE) analysis. Primers 27F and 1492R (19) were used for amplifying the 16S rRNA gene from bacterial isolates for partial sequencing by Sanger sequencing (GATC Biotech, Germany).

All PCR amplifications were performed using a Gene Amp PCR system 9700 thermocycler (Applied Biosystems). Each 50-μl PCR mixture contained 50 to 100 ng of DNA, primers (0.4 μM), deoxynucleoside triphosphates (dNTPs) (0.1 mM), *Taq* polymerase (1.25 U) (Qiagen), and 1× PCR buffer (Qiagen). For primers F341-GC and R534, thermocycling consisted of 95°C for 5 min followed by 35 cycles of 95°C for 30 s, 57°C for 30 s, and 72°C for 1.5 min, ending with 72°C for 10 min. For primers 27F and 1492R, cycling conditions consisted of 94°C for 5 min followed by 30 cycles of 94°C for 30 s, 57°C for 45 s, and 72°C for 90 s, ending with 72°C for 10 min. For primers ITS3-GC and ITS4 cycling conditions consisted of 95°C for 2 min followed by 36 cycles of 94°C for 30 s, 55°C for 30 s, and 72°C for 1 min, with a final extension at 72°C for 5 min. PCR products were analyzed on a 1% (wt/vol) 1× TAE (40 mM Tris base, 1 mM EDTA [pH 8]) agarose gel stained with ethidium bromide (10 mg ml^−1^) and viewed under UV transillumination (Alpha Innotech).

### DGGE.

Denaturing gradient gel electrophoresis (DGGE) analysis of 16S rRNA gene fragments was performed using a D-Code System (Bio-Rad) as described previously (17), with a gradient of 40 to 60%, except gels were silver stained as described elsewhere (20). DGGE of the fungal ITS region was performed as described above except that the denaturing gradient was 30 to 70%. Selected DGGE bands were excised and placed into 100 μl of nuclease-free water for storage at 4°C. DGGE bands were reamplified using either F341 and R534 primers for bacterial DNA or ITS3 and ITS4 primers for fungal DNA, purified using the GenElute PCR Clean-Up kit (Sigma-Aldrich), and sequenced by GATC Biotech (Germany). The sequences obtained were compared with public DNA database sequences using the Basic Local Alignment Search Tool (BLAST).

### 454 pyrosequencing.

DNAs extracted from filter and planktonic communities from the HMW-PAH transformation study were selected for 454 pyrosequencing. The 16S rRNA gene was amplified using the primers Bakt_341F (CCTACGGGNGGCWGCAG) and Bakt_805R (GACTACHVGGGTATCTAATCC) (21). GS FLX Titanium adaptors were at the 5′ ends of the Bakt primers, adaptor A for the forward primer (5′-CGTATCGCCTCCCTCGCGCCATCAG-3′) and adaptor B for the reverse primer (5′-CTATGCGCCTTGCCAGCCCGCTCAG-3′). In addition, each sample was given a unique reverse 10-bp primer barcode located between the B adaptor and Bakt_805R. Cycling conditions were as described in reference 21. Triplicate PCR products were pooled, purified using a QIAquick gel extraction kit (Qiagen), and analyzed using the FLX 454 Titanium sequencer (Roche) at Wageningen University (The Netherlands). The pyrosequence reads were analyzed using the QIIME pipeline and associated modules (22, 23). All sequences were checked for the presence of correct pyrosequencing adaptors, 10-base barcodes, and the 16S rRNA gene-specific primers, and any sequences containing errors in these regions were removed from analysis. Any sequences <150 bp in read length, containing 7-bp homopolymer inserts, with low-quality scores (<25), or with chimeras were also removed. The remaining reads were clustered into operational taxonomic units (OTUs) using the USearch algorithm at the 95% similarity level (24). Representative sequences from each OTU were assigned to a taxonomic group using the RDP classifier algorithm (25). The BbF filter community contained the highest number of sequences (9,140) and the BbF planktonic community the lowest (2,562), so the data were normalized to 2,562 sequences. The Pyr filter and planktonic communities were analyzed, but the number of returned reads was low, so they were not included in the analysis. Phylogenetic analysis was performed as previously described in reference 15.

### Statistical analysis.

All statistical analyses were performed using SPSS version 18.0. Analysis of DGGE profiles was performed using Primer 6 Beta, and percent similarity between communities was calculated using a binary matrix based on the presence/absence of bands, with similarity calculated using the Jaccard coefficient.

## RESULTS

### Transformation of HMW-PAHs by microbial communities from OSPW.

Two OSPW samples were used as inocula for laboratory experiments. The first, designated TPW, was aged recycled water collected from Syncrude's test pits constructed in 1993, and the second, designated 2m, was collected at a water depth of 2 m from a Suncor tailings pond. The TPW sample had a pH of 7.44, and the total organic carbon was 28.8 g liter^−1^. The 2m sample had a pH of 7.68, and the total organic carbon was 24.3 g liter^−1^. Samples were analyzed for anion and cation concentrations, and the results are presented in Table S5 in the supplemental material.

**FIG 1:**
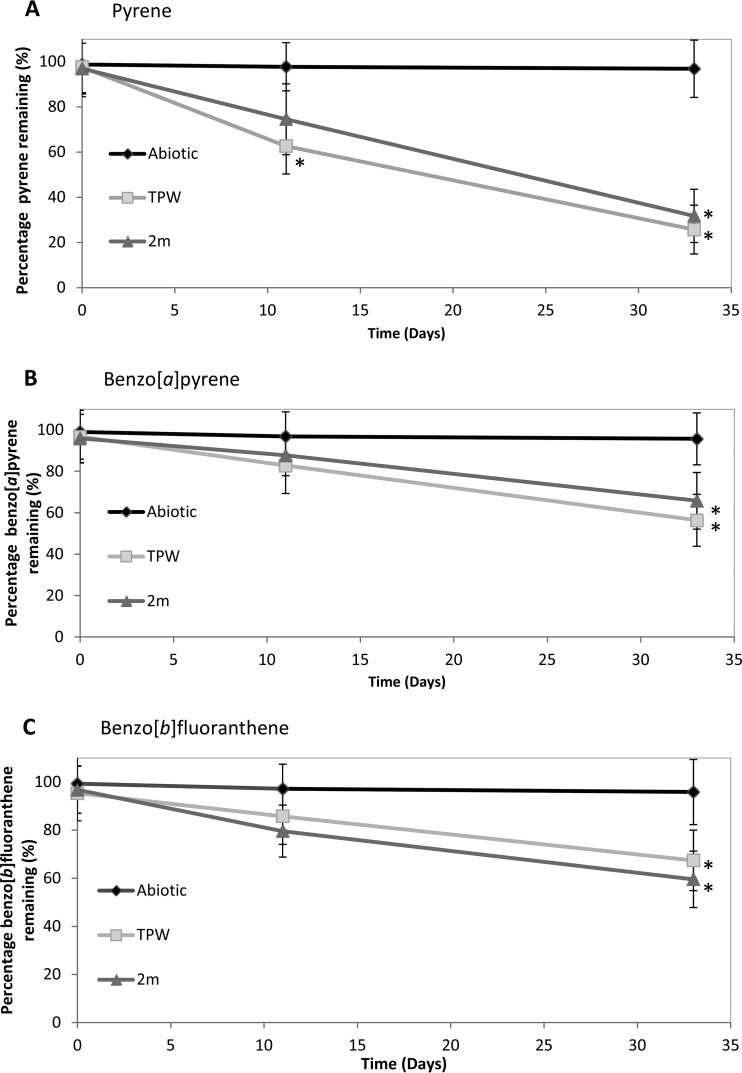
Biotransformation of HMW-PAHs by the microbial communities derived from samples TPW and 2m. Enrichments were grown on Pyr (A), BaP (B), and BbF (C). Error bars represent the standard error of the mean (*n* = 3). *, significant degradation compared to abiotic control (Mann-Whitney U test, *P* < 0.05).

The removal of Pyr, BaP, and BbF (compared with abiotic controls) by microbes in the two OSPW enrichments was investigated (Fig. 1). Twice the amount of Pyr as of BaP and BbF was transformed by both OSPW communities. By day 33, the microbial communities derived from TPW and 2m transformed 75% and 65% of Pyr, respectively (Fig. 1A). By day 11, the microbial communities from both TPW and 2m showed no significant removal of BaP or BbF (*P* > 0.05). However, by day 33, the microbial community from TPW significantly transformed 39% of BaP and 28% of BbF (*P* < 0.05), while the microbial community from 2m significantly transformed 30% of BaP and 36% of BbF (*P* < 0.05) (Fig. 1B and C).

### Metabolite production.

**FIG 2:**
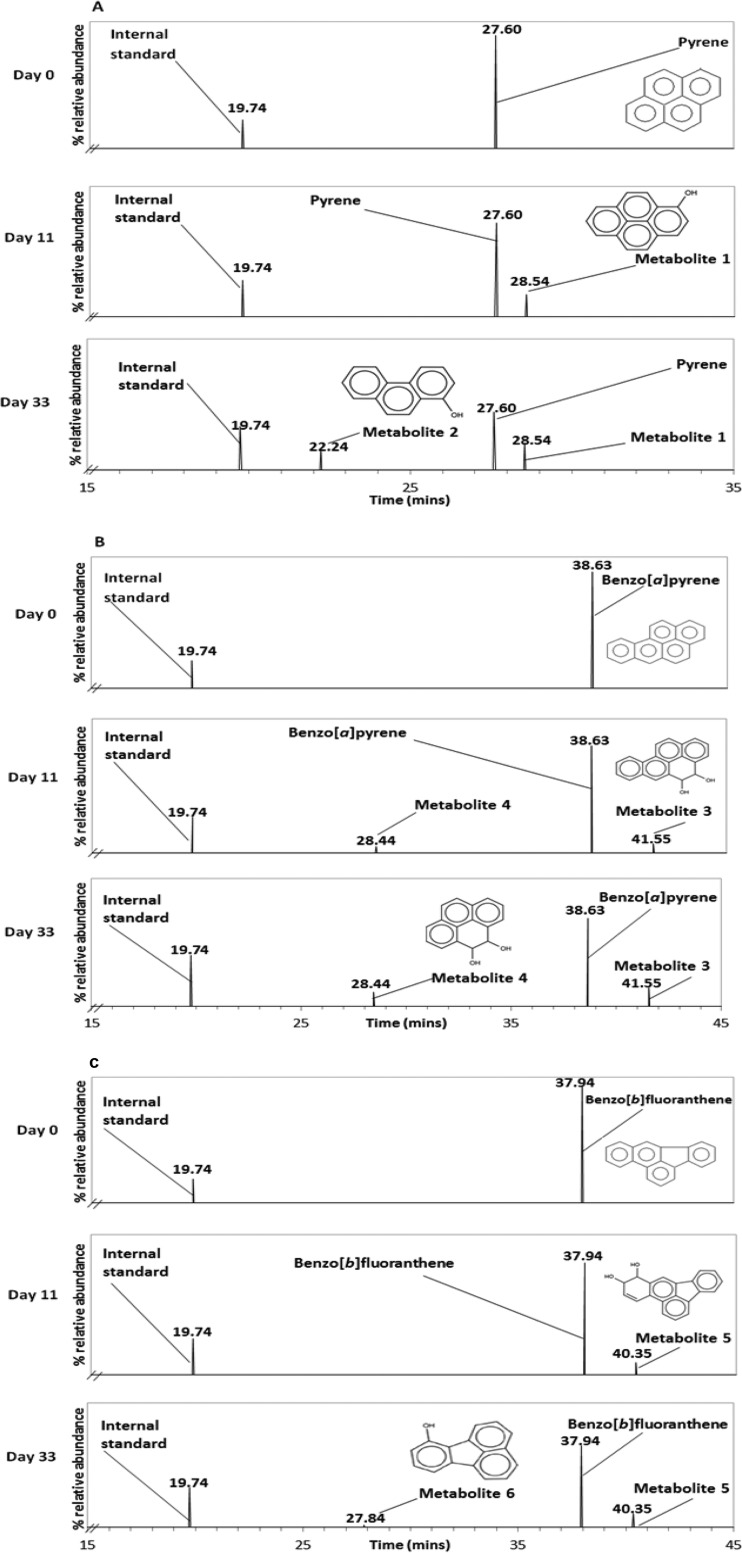
Gas chromatograms showing the transformation of the three HMW-PAHs over a 33-day incubation period by the microbial community derived from sample TPW. Enrichments were grown on Pyr (A), BaP (B), and BbF (C). There is a decrease of the Pyr (retention time [RT], 27.60 min), BaP (RT, 38.63 min), and BbF (RT, 37.94 min) peaks and the appearance of secondary and tertiary peaks associated with tentative metabolites.

**FIG 3:**
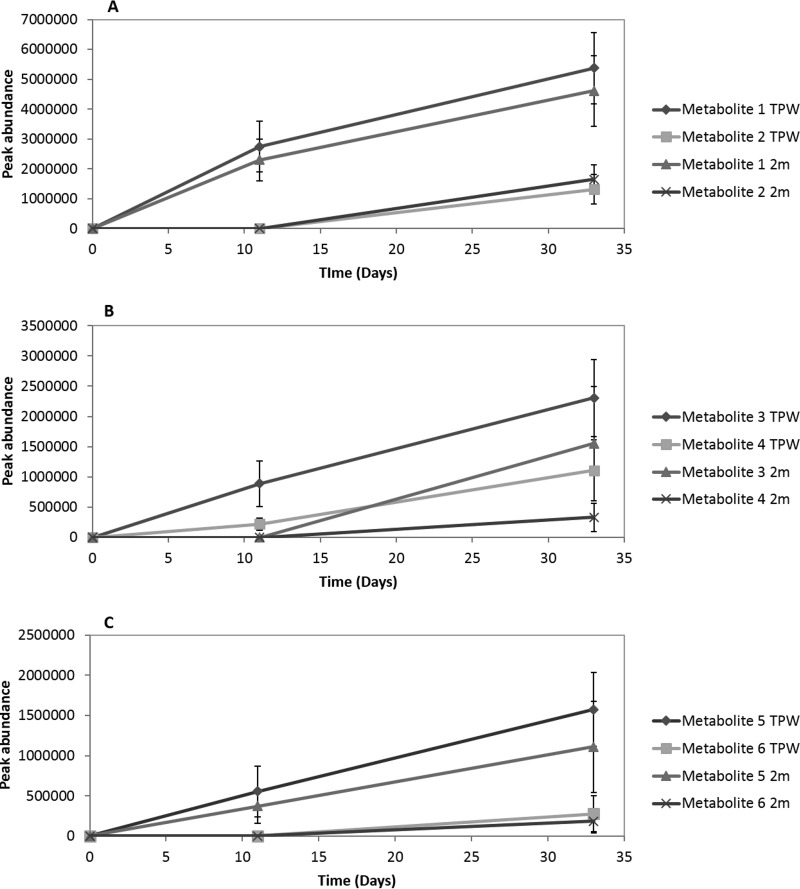
Production of metabolites tentatively identified during the transformation of Pyr (A), BaP (B), and BbF (C) by microbial communities derived from sample TPW. Error bars represent the standard error of the mean (*n* = 3).

Microbial transformation of Pyr, BaP, and BbF resulted in the production of six metabolites, two from each PAH (Fig. 2; see Fig. S1 in the supplemental material), which were identified by their mass spectra. The peak abundance of each metabolite was determined (Fig. 3). The same metabolites were detected irrespective of whether the microcosms were inoculated with TPW or 2m. During the transformation of Pyr, metabolites 1 and 2 were produced (Fig. 2A). Metabolite 1 was tentatively identified as hydroxypyrene and metabolite 2 as hydroxyphenanthrene. During the transformation of BaP, two metabolites were produced: metabolite 3 (putatively identified as 4,5-dihydroxybenzo[*a*]pyrene) and metabolite 4 (putatively identified as 4,5-dihydroxypyrene) (Fig. 2B). Metabolites 5 and 6, produced during the transformation of BbF (Fig. 2C), were tentatively identified as 9,10-dihydroxybenzo[*b*]fluoranthene and hydroxyfluoranthene, respectively. No metabolites were detected in either abiotic or killed controls.

### Bacterial and fungal community compositions in HMW-PAH enrichments.

**FIG 4:**
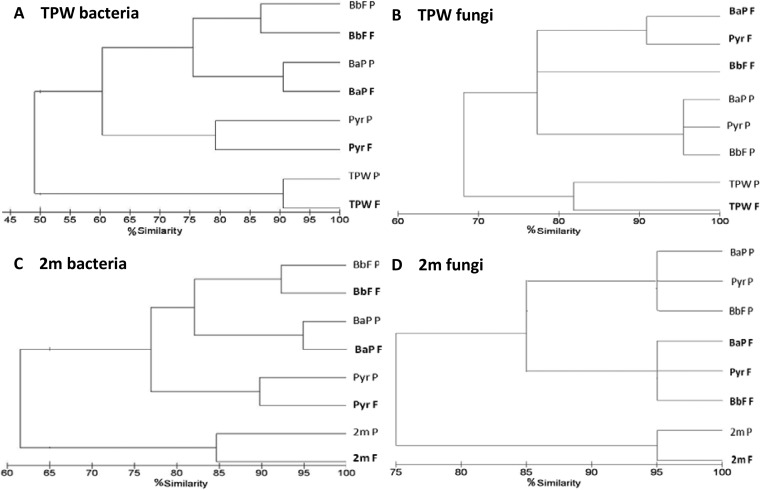
Cluster analysis illustrating similarities between bacterial and fungal community structures on day 33 of the biotransformation experiment on HMW-PAHs. (A) TPW bacterial community; (B) TPW fungal community; (C) 2m bacterial community; (D) 2m fungal community. P, planktonic community; F, filter community; TPW, TPW community with no added PAH; 2m, 2m community with no added PAH.

In order to provide an overview of any differences in bacterial and fungal community composition between treatments at day 33, DGGE analysis was performed on amplified 16S rRNA genes and the intergenic transcribed spacer (ITS) region between the 5.8S and 28S rRNA genes, respectively (Fig. 4). For enrichments derived from TPW, a significant difference (50%) (*P* < 0.05 by analysis of similarities [ANOSIM]) in the bacterial communities was observed with respect to the control (i.e., TPW inoculum with no added PAH, which had followed the same pattern of enrichment as those cultures with PAHs, but no PAH was added at any stage) (Fig. 4A). The biofilm communities on the filters were between 78 and 91% similar to the planktonic communities enriched with the same PAH. Furthermore, BaP-enriched communities were more similar to those enriched on BbF (76%) than to those enriched on Pyr (60%) (Fig. 4).

For the bacterial communities derived from sample 2m, a pattern similar to that for sample TPW was observed, with 62% similarity in composition between the control communities (i.e., with no added PAH) and those enriched on HMW-PAHs (Fig. 4C). The biofilm communities were 85 to 95% similar to planktonic communities for each PAH.

For enrichments derived from both samples TPW and 2m, the fungal communities were less diverse than the bacterial communities based on the number of DGGE bands observed (see Fig. S2B and S3B in the supplemental material). For TPW-derived fungal communities, there was 68% similarity between the control community and those communities enriched on HMW-PAHs (Fig. 4B). In contrast to the bacterial community, the fungal communities did not cluster according to the added substrate. The biofilm communities enriched on the filters containing Pyr and BaP were approximately 92% similar to each other, with the BbF filter community being 77% similar. Planktonic communities were 95% similar to each other (regardless of PAH). A similar trend was observed with the 2m fungal communities (Fig. 4D), whereby the biofilm communities enriched on the filters were 95% similar to each other and planktonic communities had 95% similarity to each other regardless of PAH.

### Sequencing of the 16S rRNA gene to identify bacteria in HMW-PAH-transforming enrichments derived from sample TPW.

**FIG 5:**
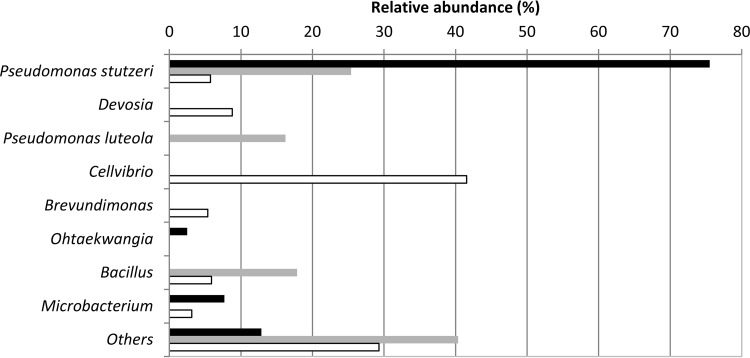
Filter and planktonic bacterial communities from sample TPW enriched on BbF and analyzed by 454 pyrosequencing of the 16S rRNA genes. Bacterial genera compromising >3% of the total community from the BbF filter (black bars), BbF planktonic (gray bars), and no-PAH control (white bars) communities are shown. Others, sum of abundances for genera representing <3% of the community composition.

Communities derived from TPW enrichments were characterized by 454 pyrosequencing of the bacterial 16S rRNA gene (Fig. 5), in order to provide a more in-depth view of the bacterial communities present in selected samples. Amplified DNAs from triplicate samples were pooled (as replicates had very similar DGGE profiles [see Fig. S2A in the supplemental material]). Clustering of quality-controlled sequences at the 95% similarity level, and comparison of OTUs representing >3% of the total community were performed to identify the main changes in bacterial community composition. All incubations were analyzed by 454 pyrosequencing, but the numbers of reads were low (<100) in Pyr samples and the no-added-PAH planktonic control, so they were not included in the analysis.

Compared with the no-PAH-filter control, an OTU very similar to Pseudomonas stutzeri increased 4-fold in the BbF planktonic community and 13-fold in the BbF filter community, constituting 76% of the OTUs in the latter (Fig. 5). In addition to Pseudomonas stutzeri, in the BbF filter community, 8% of the OTUs were similar to a Microbacterium sp., while in the BbF planktonic community, 16% of the OTUs were similar to Pseudomonas luteola and 18% were similar to a Bacillus sp. In contrast, addition of BbF selected against Cellvibrio, Devosia, and Brevundimonas, which constituted <1% of the BbF filter and planktonic communities and 42%, 9%, and 5% of the no-PAH community, respectively (Fig. 5). A detailed summary of the genera comprising >1% of the total community from the pyrosequenced samples is provided in Table S1 in the supplemental material.

Sequence analysis of the DGGE bands that differed in abundance gave a broad comparison of the differences in the main taxa between treatments (see Fig. S2A and S3A in the supplemental material). Bacterial 16S rRNA gene sequence analysis from the TPW enrichment (see Fig. S2A and Table S2 in the supplemental material) revealed that the planktonic community grown on Pyr had three specific DGGE bands, which had high 16S rRNA gene sequence identities to Curtobacterium (98%) Arthrobacter (99%), and Phenylobacterium (98%). The Pyr filter community contained one specific DGGE band, which had a high 16S rRNA gene sequence identity (98%) to Bacillus lentus. Both the filter and planktonic communities enriched on either BaP or BbF contained two bands, which had high 16S rRNA gene sequence identities to Pseudomonas stutzeri (99%) and a Microbacterium sp. (99%), respectively (see Fig. S2A in the supplemental material).

A number of different species were identified in the 2m bacterial community. Two bands specific to the Pyr filter community had a high 16S rRNA gene sequence identity to a Streptomyces sp. (99%) and a Micromonospora sp. (99%), while two bands specific to the Pyr planktonic community had a high 16S rRNA gene sequence identity to Paracoccus aminovorans (98%) and Pseudomonas spp. (98%) (see Fig. S3A in the supplemental material).

Sequence analysis of selected fungal DGGE bands showed that all the TPW PAH-enriched filter communities (but not the no-PAH controls) contained one specific DGGE band, which had a high ITS sequence identity (99%) to Cladosporium cladosporioides (see Fig. S2B and Table S3 in the supplemental material). In the 2m fungal community, one band, specific to the BbF filter community, also had ITS sequence identity to Cladosporium spp. (94%) (see Fig. S3B and Table S3 in the supplemental material).

### Isolation of bacteria from HMW-PAH enrichments.

Five pure bacterial cultures were isolated (see Table S3 in the supplemental material) from HMW-PAH-transforming communities. Strains A1, A6, and A13 from filters containing Pyr, BaP, and BbF, respectively, had 99% 16S rRNA gene sequence similarity to members of the genus Streptomyces. Strains A3 and A7, isolated from the culture broth from enrichments containing Pyr and BaP sorbed to filters, had a high 16S rRNA gene sequence similarity to Microbacterium hydrocarbonoxydans (99%). No growth was observed with any of the isolates on plates supplemented with acetone only.

### Phylogenetic analysis.

**FIG 6:**
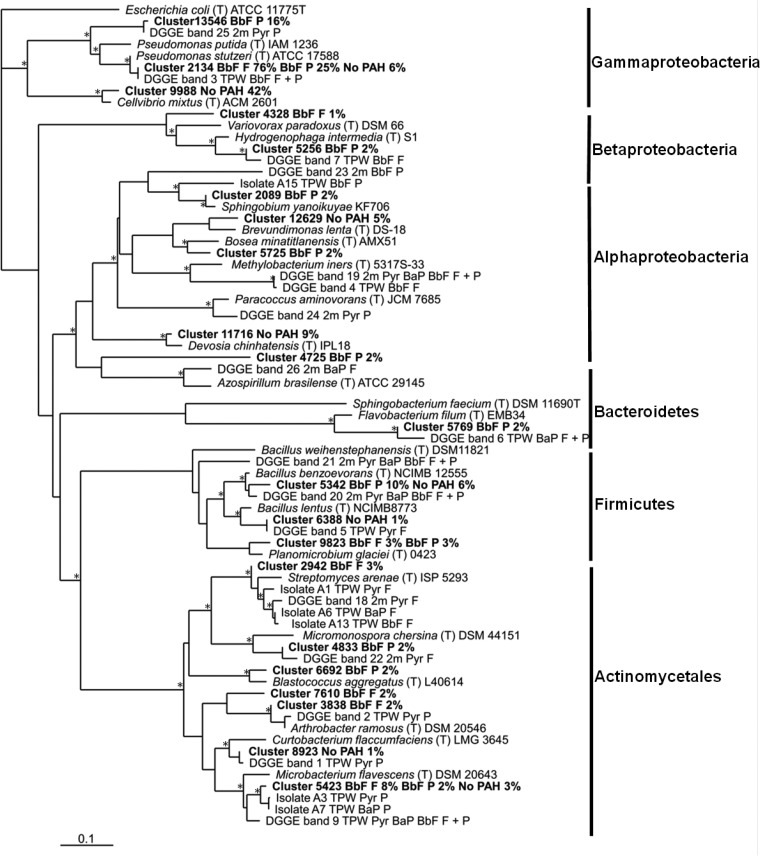
Phylogenetic analysis of selected 16S rRNA gene sequences from isolates, pyrosequencing, and DGGE from the HMW-PAH-degrading communities. For each OTU, the most closely related sequences from GenBank are also indicated. Sequence analysis was performed using the neighbor-joining method. Bootstrap values represent percentages from 100 replicates of the data, and those of >80% are shown by an asterisk. The scale bar indicates 0.01 substitution per nucleotide base. Pyr, pyrene enrichments; BaP, benzo[*a*]pyrene enrichments; BbF, benzo[*b*] fluoranthene enrichments; F, filter; P, planktonic.

Phylogenetic analysis was performed on the cultured (six representative isolates) and noncultured bacteria from the HMW-PAH enrichments: 20 representatives of the most abundant pyrosequence OTUs (i.e., those comprising >3% of the total community) and 17 DGGE bands (Fig. 6). All sequences grouped with sequences from Actinobacteria, Firmicutes, Bacteroidetes, or *Alpha*-, *Beta*-, or Gammaproteobacteria. Within the Actinomycetales, Streptomyces-related sequences from isolates, DGGE bands, and pyrosequences all derived from attached bacteria. Cluster 2942 (Fig. 6) (Streptomyces) represented 3% of the BbF filter community and <1% of the BbF planktonic and no-PAH control communities. The isolates related to Microbacterium spp., although cultured from the planktonic phase, were also attached to the filters as judged by sequences being detected from filters by DGGE. Furthermore, the highly abundant pyrosequence cluster 2134 (Fig. 6) from the BbF filter community and the DGGE bands from the same enrichment were similar to Pseudomonas stutzeri and identical to each other. Other DGGE and isolate sequences from the Alphaproteobacteria clustered with Methylobacterium and Sphingobium.

## DISCUSSION

Filters were used to imitate PAH sorption to organic matter and other particles in aquatic environments. It was hypothesized that hydrophobic filters with sorbed HMW-PAHs would select for microbes that specialize in surface adhesion, whereby attached PAH-degrading microbes would have a selective advantage over those in the planktonic phase, by reducing the distance between cells and the carbon source and encouraging PAH degradation (13, 26). The enrichment of microorganisms attached to filters containing a sorbed HMW-PAH implicates them as a potential degrading species.

OSPW were found to harbor active communities, capable of the removal and biotransformation of HMW-PAHs. In addition, hydrophobic filters containing sorbed HMW-PAHs selected for a different microbial community composition (especially with the fungal community) than microbes in the planktonic phase. Bacterial communities were also influenced by the specific HMW-PAH used for enrichment, while fungal communities were not. To date, there is little information on the composition of the microbial communities involved in the biotransformation of HMW-PAHs in aquatic environments, specifically with regard to freshwater fungal species. Despite the fact that PAHs are known to be abundant in oil sands (5, 6), attention has focused primarily on the acutely toxic naphthenic acids (7). To our knowledge, this is the first report to characterize the microbial communities from OSPW with the ability to transform HMW-PAHs.

We found that Pyr was more readily transformed than both BaP and BbF. Hydroxypyrene was detected during the metabolism of Pyr, as has been shown in bacterial (27, 28, 29) and fungal (10, 30, 31, 32) cultures. During the transformation of BaP, the metabolite 4,5-dihydroxybenzo[*a*]pyrene (metabolite 3) was produced. It has previously been detected in cultures of Mycobacterium vanbaalenii PYR-1 degrading BaP (33). Other hydroxylated intermediates of BaP, including 9,10-dihydroxybenzo[*a*]pyrene, an isomer of metabolite 3, have also been proposed in bacterial (34) and fungal (31) biodegradation pathways. The metabolite 4,5-dihydroxypyrene (metabolite 4 in the present study) is a commonly detected metabolite during Pyr degradation in aquatic environments by both bacteria and fungi (31, 35, 36, 37, 38). Thus, metabolites typically associated with aerobic degradation of Pyr and BaP (39) were detected. Here, we tentatively identified 9,10-dihydroxybenzo[*b*]fluoranthene and hydroxyfluoranthene as metabolites of BbF transformation (see Fig. S1E and F in the supplemental material). A number of bacterial biotransformation products of BbF have also been previously identified, including hydroxylated intermediates (40), but no specific fungal biodegradation products of BbF have been described previously. Both dihydroxybenzo[*k*]fluoranthene and benzo[*k*]fluoranthenedihydrodiol have also been proposed as hypothetical intermediates of benzo[*k*]fluoranthene (a closely related isomer of BbF) metabolism by Sphingobium sp. strain KK22 (41).

Distinct bacterial communities were selected during the biotransformation of different PAHs, indicating that there are some taxon-specific adaptations for the uptake of different parent compounds or their metabolites. In the present study, 16S rRNA sequences related to Pseudomonas stutzeri were found in cultures containing BbF, which suggests that this species may be able to transform HMW-PAHs in OSPW, particularly since sequences from these microorganisms constituted 76% of the bacterial BbF filter community, 25% of the BbF planktonic community, and only 6% of the no-PAH control community. A number of Pseudomonas stutzeri strains have had their genomes sequenced, including the naphthalene-degrading strain AN10 (42). Pseudomonas stutzeri has broad phenotypic and genotypic diversity, with the potential to form biofilms (43, 44). Furthermore, Pseudomonas stutzeri strain T102 demonstrated more rapid naphthalene degradation when grown as a biofilm than when in planktonic cultures. It was suggested that the high degradation activity may come from superactivated cells that detached from the biofilms, as the expression levels of *nahAc* (naphthalene dioxygenase large subunit) were significantly higher in detached cells than in either planktonic cells or biofilms (45).

In addition to enriching for Pseudomonas stutzeri, filters promoted differential growth of filamentous organisms, including the fungi and certain species of Actinomycetales. For example, 8% of the pyrosequences from the BbF filter community belonged to the genus Microbacterium, which has previously been associated with the degradation of phenanthrene and pyrene (46) and crude oil (47). Filters containing HMW-PAHs were enriched for Streptomyces spp. (detected as pyrosequencing cluster 2942 [Fig. 6], DGGE band 18, and three isolates). Previous studies using ^13^C-labeled Pyr identified Streptomyces and other actinobacteria as PAH degraders (48). Streptomyces coelicolor has also been shown to produce broad-spectrum multicopper oxidases with activity against a variety of PAH substrates (49). In the absence of the HMW-PAH on the filter (as in a traditional liquid enrichment cultures, where the carbon source is provided in solution or applied to the glass), these actinobacterial species may not have been identified as potential HMW-PAH degraders. The enrichment of Actinomycetales species in filter-attached communities was also found by Bastiaens et al. (14), who demonstrated that PAH enrichments using sorbing filters led to the isolation of Mycobacterium spp., whereas liquid cultures yielded Sphingomonas spp. While Bastiaens et al. (14) focused on investigating bacterial isolates, using two separate methods for enrichment from soil, the present study examined both bacterial and fungal community compositions, enriched on different PAHs, from an aquatic environment and used an enrichment method that facilitated direct comparisons between biofilm and planktonic communities. It should be noted that in the present study, differences were observed between the isolate sequences and those recovered from DGGE bands and pyrosequencing libraries. It is possible that the microorganisms identified in the sequencing libraries and DGGE gels were either unable to grow on the solid agar plates or grew very slowly and were not detected.

PCR-DGGE analysis of the fungal communities identified that members were not PAH specific; rather, they tended to be found either attached to filters only or in suspension in the planktonic phase only. For example, ligninolytic, Cladosporium cladosporioides (band 17) was detected only on filters and was enriched in the presence of all HMW-PAHs but was not detected in the no-PAH control (see Fig. S2 in the supplemental material). Cladosporium species from a number of terrestrial environments have been shown to degrade a variety of PAHs with more than three rings (31). To date, a single species, Cladosporium herbarum, has been isolated from an aquatic environment, specifically a PAH-contaminated river sediment (50). Furthermore, pure cultures of Cladosporium sphaerospermum have been shown to degrade BaP, with laccase activity detected during degradation. However, there was no correlation between laccase activity and BaP loss, implying that the laccase provides an indirect mechanism of degradation (51). In contrast to finding Cladosporium on filters, we detected nonligninolytic Penicillium spp. in the planktonic phases of BaP, Pyr, and BbF enrichments (bands 13 and 15) but not in the no-PAH control. Penicillium spp. from a variety of terrestrial environments have been isolated and shown to degrade Pyr and BaP to various degrees (reviewed in reference 52) in aquatic environments. For example, three Penicillium species isolated from PAH-contaminated sediments were shown to degrade Pyr (50).

PAHs selected for a distinct fungal community compared with the no-PAH control. However, we cannot say whether the fungi grew using the PAH as a source of carbon and energy, nor are there any uniquely fungal metabolites that would definitively implicate them in the initial oxidation of the parent compounds. The fact that the fungal community composition was driven more by whether it was in the planktonic or solid phase than by the specific PAH in the medium may be explained by the fact that fungi tend to produce nonspecific extracellular oxidative enzymes (31). A wide range of lignin peroxidases, manganese peroxidases, and laccases have been identified from ligninolytic fungi, such as Cladosporium, which are involved in the degradation of Pyr and BaP as well as many other HMW-PAHs (reviewed in reference 31). The same capabilities occur in freshwater fungi, particularly the production of laccase-type enzymes (53, 54). With this in mind, it is possible that the initial attack on HMW-PAHs by fungal exoenzymes is more likely than attack by bacterial intracellular, and cofactor-requiring, enzymes, because they diffuse to the highly immobile HMW-PAHs (12). However, the nonligninolytic species Penicillium janthinellum has been shown to degrade Pyr in a reaction catalyzed by a cytochrome P450 monooxygenase (30). If the fungi did not use PAHs for growth, they could have used metabolites produced by bacteria or their dead cells.

In conclusion, we demonstrated that OSPW harbors microbial communities with the capacity to metabolize HMW-PAHs. During the transformation of HMW-PAHs, a number of metabolites were produced and tentatively identified as the hydroxylated intermediates of the parent compound. In addition, we demonstrated that the provision of suitable surfaces that encourage PAH sorption and microbial adhesion select for fungal and bacterial communities with the potential for HMW-PAH metabolism that may have been overlooked previously by the use of liquid enrichments. We showed that a major factor determining the fungal community composition was whether it was present in the planktonic phase or attached to filters. In contrast, despite the fact that some bacteria (e.g., Pseudomonas stutzeri and Streptomyces spp.) had a preference for growing on filters, the bacterial community composition was selected primarily by the PAH serving as the carbon source. This suggests that diverse bacterial communities would be required to degrade a range of HMW-PAHs. Future work should focus on elucidating the interactions between bacterial and fungal members of these consortia to improve the biodegradation rates of HMW-PAHs, an important suite of pollutants that are often overlooked in tailings ponds containing oil sand waste.

## Supplementary Material

Supplemental material
