# Supplementary material for: Biofilm and Planktonic Bacterial and Fungal Communities Transforming High-Molecular-Weight Polycyclic Aromatic Hydrocarbons
Source: Appl Environ Microbiol. 2016 Apr 4;82(8):2288–99. doi: 10.1128/AEM.03713-15 (PMC4959499; doi:10.1128/AEM.03713-15)
Supplement: Supplemental material [file supp_82_8_2288__index.html]

Biofilm and Planktonic Bacterial and Fungal Communities Transforming High-Molecular-Weight Polycyclic Aromatic Hydrocarbons — Supplemental material 

# Biofilm and Planktonic Bacterial and Fungal Communities Transforming High-Molecular-Weight Polycyclic Aromatic Hydrocarbons

## Supplemental material

- Supplemental file 1 -

  Composition of BbF-degrading filter, planktonic, and control communities from 454 pyrosequencing analysis (Table S1), Blastn analysis of the 16S rRNA gene sequences obtained from excised bacterial DGGE bands (Table S2), Blastn analysis of the ITS sequences obtained from excised fungal DGGE bands (Table S3), Blastn analysis of 16S rRNA gene sequences obtained from isolates (Table S4), anion and cation analysis of TPW and 2m OSPW samples (Table S5), mass spectra of metabolites produced during degradation of HMW-PAHs (Fig. S1), composite image of DGGE gel of the TPW sample, demonstrating microbial community structure change under the selective pressure of HMW-PAHs (Fig. S2), and DGGE gel from the enrichment of 2m sample, demonstrating microbial community structure change under the selective pressure of HMW-PAHs (Fig. S3).

  PDF, 473K
