# Supplementary material for: Biofilm and Planktonic Bacterial and Fungal Communities Transforming High-Molecular-Weight Polycyclic Aromatic Hydrocarbons
Source: Appl Environ Microbiol. 2016 Apr 4;82(8):2288–99. doi: 10.1128/AEM.03713-15 (PMC4959499; doi:10.1128/AEM.03713-15)
Supplement: Supplemental material [file AEM.03713-15_zam999117062so1.pdf]

Characterisation of biofilm and planktonic bacterial and fungal communities biotransforming high molecular weight polycyclic aromatic hydrocarbons

SUPPLEMENTRY INFORMATION

Running Title: Biotransformation of HMW-PAHs by microbial communities

Benjamin D Folwell[1,2], Terry J McGenity[1*], and Corinne Whitby[1]*

[1]School of Biological Sciences, University of Essex, Wivenhoe Park, Colchester, CO4 3SQ, UK.

[2]Present address: Oil Plus Ltd., Dominion House, Kennet Side, Newbury, RG14 5PX, UK.

*Corresponding authors:

tjmcgen@essex.ac.uk, Tel: +44 1206 872535 Fax +44 1206 872592

cwhitby@essex.ac.uk, Tel: +44 1206 872062 Fax +44 1206 872592

Supplementary Table S1 Composition of BbF degrading filter, planktonic and control communities from 454 pyrosequencing analysis.

Supplementary Table S2 Blast*n* analysis of the 16S rRNA gene sequences obtained from excised bacterial DGGE bands.

Supplementary Table S3 Blast*n* analysis of the ITS sequences obtained from excised fungal DGGE bands.

Supplementary Table S4 Blast*n* analysis of 16S rRNA gene sequences obtained from isolates.

Supplementary Table S5 Anion and cation analysis of TPW and 2m OSPW samples (ND: Not detected).

Supplementary Fig. S1. **Mass spectra of metabolites produced during degradation of HMW-PAHs.** Mass spectra of metabolites produced during the degradation of Pyr tentatively identified as hydroxypyrene (A) (molecular mass of 218 amu) and tentatively identified as hydroxyphenanthrene (B) (molecular mass of 178 amu). Mass spectra of metabolites produced during the degradation of BaP (C) tentatively identified as 4-5, dihydroxybenzo[*a*]pyrene (C) (molecular mass of 268 amu) and tentatively identified as 4-5, dihydroxypyrene (D) (molecular mass of 234 amu). Mass spectra of metabolites 5 produced during the degradation of BbF tentatively identified as 9,10 dihydrobenzo[*b*]fluoranthene (E) (molecular mass of 268) amu and tentatively identified as hydroxfluoranthene (F) (molecular mass of 218 amu).

Supplementary Fig. S2 Composite image of DGGE gel of the TPW sample, demonstrating microbial community structure change under the selective pressure of HMW-PAHs. Bacterial community (A) and fungal community (B). For all samples F = filter community and P = planktonic community. % indicates the different DGGE gradients used. Numbers refer to bands identified in Tables S2 and S3.

Supplementary Fig. S3 DGGE gel from the enrichment of 2m sample, demonstrating microbial community structure change under the selective pressure of HMW-PAHs. Bacterial community (A) and fungal community (B). For all samples F = filter community and P = planktonic community. Numbers refer to bands identified in Tables S2 and S3.

Supplementary Table S1 Composition of BbF degrading filter, planktonic and control communities from 454 pyrosequencing analysis.

**Enrichment**			**TPW (no added PAH)**	**BbF Filter**	**BbF planktonic**
Number of sequences retrieved			4082	9140	2562
Class	Genus	OTU	Percentage of community		
Alphaproteobacteria	*Devosia*	11716	**7.8**	0	1.5
	Bosea	5725	0.2	**2.2**	1.9
	Brevimondonas	12629	**4.4**	0.3	0
	Rhodospirillum	3177	0	1	0.8
	Caulobacter	1286	1	0	0.1
	Sphingobium	2089	1.2	0	2
	Rubellimicrobium	7544	0.5	0	1
	Azospirillum	4725	0.58	0.1	**2.1**
	Parvibaculum	6386	1.2	0.1	0.4
Betaproteobacteria	*Variovorax*	4328	0.2	1.2	0.4
	Hydrogenophaga	5256	1.3	0.1	1.3
	Massilia	14689	1	0.2	0.3
	Oxalicibacterium	10978	0.2	0.2	1
Gammaproteobacteria	*Pseudomonas*	2134	**5.75**	**75.1**	**25.4**
	Pseudomonas	13546	0.2	0	**16.22**
	Cellvibrio	9988	**41.2**	0	0.12
Bacteroidetes	*Flavobacterium*	5769	1.3	0	2.1
	Sediminibacterium	8313	1.8	0.2	0.4
	Ohtaekwangia	320	0	1.1	0.48
Firmicutes	*Bacillus*	5342	**6.4**	1.1	**17.8**
	Bacillus	6388	1.4	0.1	0.2
	Planomicrobium	9823	1.2	0.2	2.4
Actinomycetales	*Streptomyces*	2942	0.1	**3.1**	0.4
	Micromonospora	4833	0.8	0.1	**2.2**
	Blastococcus	6692	0.8	0.1	1.7
	Arthrobacter	7610	0.8	2.1	0.7
	Arthrobacter	3838	0.6	**2.2**	0.4
	Curtobacterium	8923	1.4	0.5	0.1
	Microbacterium	5423	3.1	**7.1**	1.7
	Kibdelosporangium	1049	0.2	0.1	1.1
	Cellulomonas	10312	0.6	0.1	1.3
Others	-	-	12.77	1.4	12.48

Supplementary Table S2 Blast*n* analysis of the 16S rRNA gene sequences obtained from excised bacterial DGGE bands.

DGGE band	Compound	Filter (F) or Planktonic (P)	Closest match from Blast*n*	Sequence length (bp)	% 16S rRNA gene sequence similarity	Environment from which closest match was derived	Genbank accession number of most closely related sequence
			**Bacterial TPW HMW-PAH amended community**				
1	Pyr	P	*Curtobacterium* spp.	195	98	Sediment	NR_104839.1
2	Pyr	P	*Arthrobacter* spp.	207	99	Creosote polluted soil	NR_074770.1
3	BbF	F + P	*Pseudomonas stutzeri*	186	99	Soil	NR_103934.1
4	BbF	F	*Methylobacterium* spp.	190	95	Soil	NR_044129.1
5	Pyr	F	*Bacillus lentus*	207	98	Sediment	NR_040792.1
6	BaP	F + P	*Flavobacterium* spp.	199	96	Activated sludge	NR_043767.1
7	BbF	F	*Hydrogenophagea* spp.	204	99	Activated sludge	NR_029024.1
8	Pyr	P	*Phenylobacterium* spp.	189	98	Aquifer	NR_029117.1
9	Pyr, BaP, BbF	F + P	*Microbacterium* spp.	206	99	Oil-contaminated soil	NR_074770.1
			**Bacterial 2m HMW-PAH amended community**				
18	Pyr	F	*Streptomyces violaceus*	185	98	Soil	NR_041115.1
19	BbF	F	*Methylobacterium iners*	207	95	Soil	NR_044129.1
20	BbF	F + P	*Bacillus* spp.	193	98	Silage	NR_028709.1
21	BbF	F + P	*Bacillus odysseyi*	208	97	Soil	NR_025258.1
22	Pyr	F	*Micromonospora* spp.	179	99	Freshwater	NR_074416.1
23	BbF	P	Sphingomonadacae spp.	186	93	Sediment	NR_044320.1
24	Pyr	P	*Paracoccus aminovorans*	197	98	Soil	NR_025857.1
25	Pyr	P	*Pseudomonas* spp.	216	98	Soil	KF454850.1
26	BaP	F	*Azospirillum* spp.	193	92	Soil	NR_042845.1

Supplementary Table S3 Blast*n* analysis of the ITS sequences obtained from excised fungal DGGE bands.

DGGE band	Compound	Filter (F) or Planktonic (P)	Closest match from Blast*n*	Sequence length (bp)	% 16S rRNA gene sequence similarity	Environment from which closest match was derived	Genbank accession number of most closely related sequence
			**Fungal TPW HMW-PAH amended community**				
12	BaP	F	*Eurotiomycetes* spp.	268	92	Soil	JQ761474.1
13	BaP	P	*Penicillium* spp*.*	282	99	Sediment	JN032680.1
14	Pyr	P	*Davidiella tassiana*	261	99	Sediment	JX535143.1
15	Pyr, BaP, BbF	P	*Penicillium chrysogenum*	279	93	Soil	JF440603.1
16	Pyr, BaP, BbF	F + P	*Dothideomycetes* spp.	257	96	Soil	JQ758669.1
17	Pyr, BaP, BbF	F	*Cladosporium cladosporioides*	281	99	Soil	JX981454.1
			**Bacterial 2m HMW-PAH amended community**				
29	Pyr	F	Uncultured fungal clone	232	93	Arctic soil	FJ237052.1
30	BbF	F	*Cladosporium* spp*.*	263	94	Soil	HF952649.1
31	BaP	F	*Ustilago avenae*	259	99	Soil	AY740063.1
32	Pyr	P	*Alternaria* spp.	271	98	Soil	JX909350.1
33	Pyr, BaP, BbF	F + P	*Phaeosphaeriopsis* spp.	269	92	Sediment	KC507202.1
34	BaP	P	*Xenobotryosphaeria* spp.	258	92	Soil	KF251279.1

Supplementary Table S4 Blast*n* analysis of 16S rRNA gene sequences obtained from isolates.

Isolate	Carbon	Filter or	Closest match	% 16S rRNA	Genbank accession number of

ID	source	planktonic		sequence identity	most closely related sequence
A1	Pyr	Filter	*Streptomyces* spp. ISP 5310	99	NR_042101.2
A3	Pyr	Planktonic	*Microbacterium hydrocarbonoxydans*	99	NR_042263.1
A6	BaP	Filter	*Streptomyces violaceolatus* strain NBRC 13103	99	NR_041115.1
A7	BaP	Planktonic	*Microbacterium hydrocarbonoxydans*	99	NR_042263.1
A13	BbF	Filter	*Streptomyces violaceolatus* strain ISP 5205	99	NR_114831.1

Supplementary Table S5 Anion and cation analysis of TPW and 2m OSPW samples (ND: Not detected).

Ion	Concentration in OSPW TPW sample (μ mol L^{-1})	Concentration in OSPW 2m sample (μ mol L^{-1})
Fluoride	131.3	490
Formate	14.2	349.3
Acetate	ND	470.4
Chloride	7164.5	14334.9
Bromide	3.3	9.1
Nitrate	2.4	14
Sulfate	1264	1419.5
Phosphate	ND	18
Lithium	45.1	121.2
Sodium	28290.7	36136.6
Potassium	475	3479.3
Magnesium	728.9	1970
Calcium	512.6	2174.3
Strontium	11.5	13.4
Barium	10.32	94.5

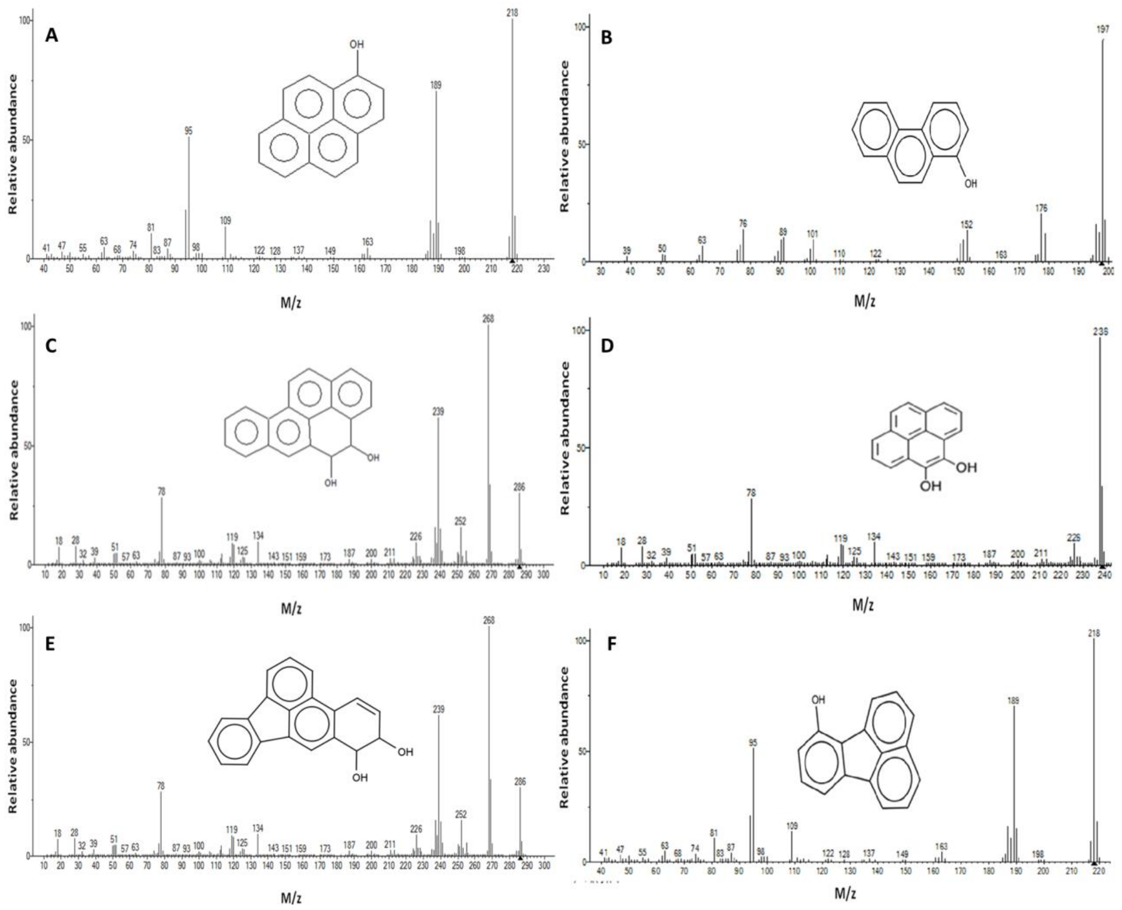

Supplementary Fig. S1. **Mass spectra of metabolites produced during degradation of HMW-PAHs.** Mass spectra of metabolites produced during the degradation of Pyr tentatively identified as hydroxypyrene (A) (molecular mass of 218 amu) and tentatively identified as hydroxphenanthrene (B) (molecular mass of 197 amu). Mass spectra of metabolites produced during the degradation of BaP (C) tentatively identified as 4-5, dihydroxybenzo[*a*]pyrene (C) (molecular mass of 268 amu) and tentatively identified as 4-5, dihydroxypyrene (D) (molecular mass of 234 amu). Mass spectra of metabolites 5 produced during the degradation of BbF tentatively identified as 9,10 dihydrobenzo[*b*]fluoranthene (E) (molecular mass of 268) amu and tentatively identified as hydroxfluoranthene (F) (molecular mass of 218 amu).

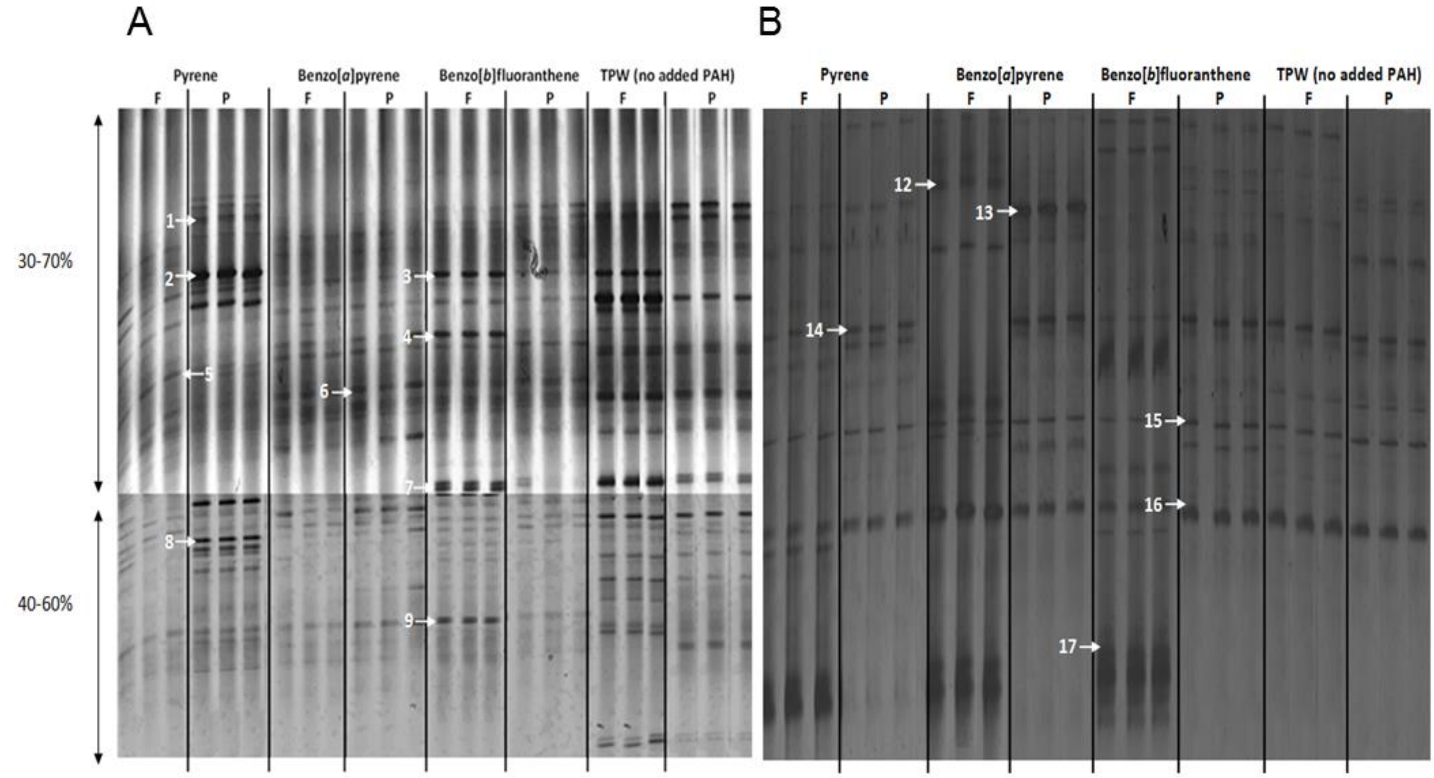

Supplementary Fig. S2 Composite image of DGGE gel of the TPW sample, demonstrating microbial community structure change under the selective pressure of HMW-PAHs. Bacterial community (A) and fungal community (B). For all samples F = filter community and P = planktonic community. % indicates the different DGGE gradients used. Numbers refer to bands identified in Tables S2 and S3.

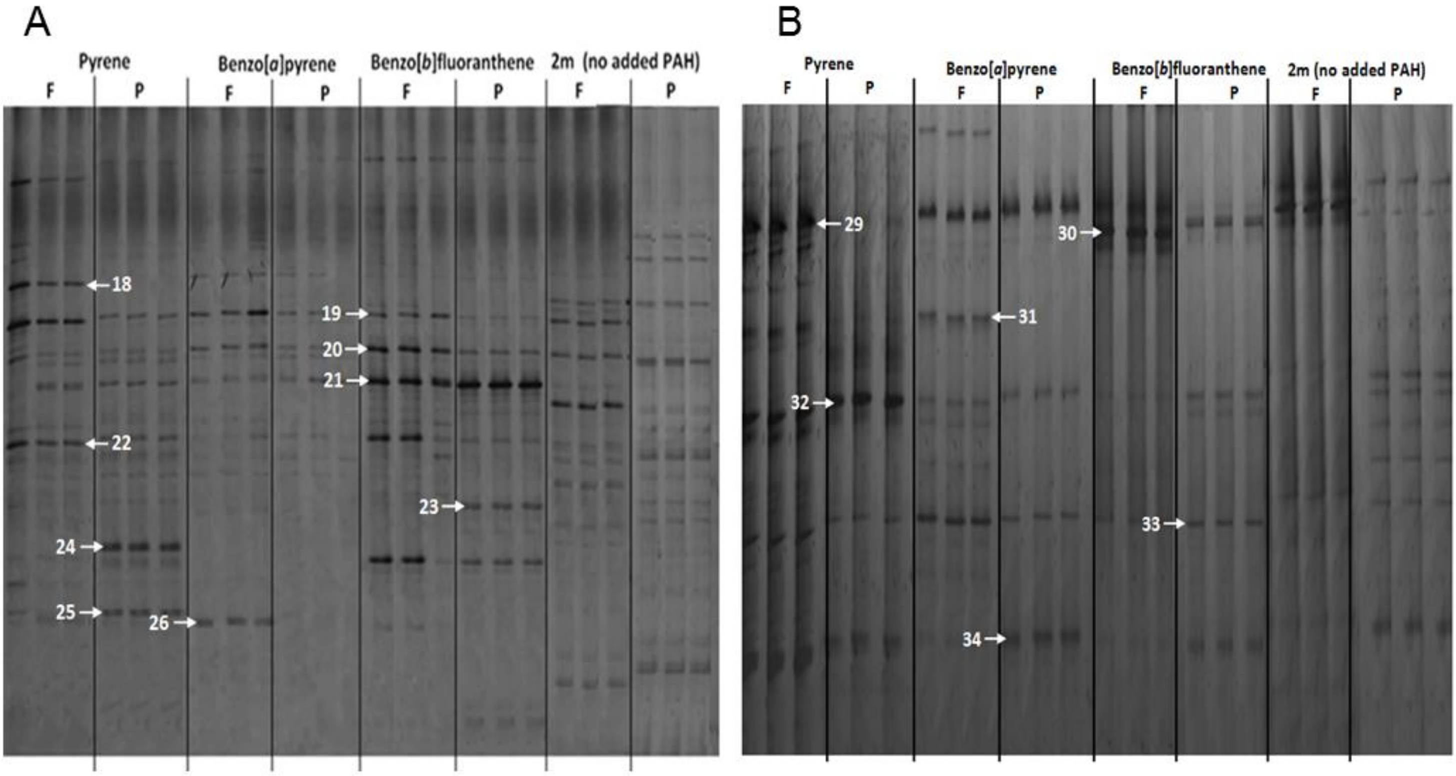

Supplementary Fig. S3 DGGE gel from the enrichment of 2m sample, demonstrating microbial community structure change under the selective pressure of HMW-PAHs. Bacterial community (A) and fungal community (B). For all samples F = filter community and P = planktonic community Numbers refer to bands identified in Tables S2 and S3.
